# Breast Cancer Among Indigenous and Non-Indigenous Women at the Mexican Teachers' Cohort

**DOI:** 10.1200/GO.22.38000

**Published:** 2022-05-05

**Authors:** Liliana Gómez-Flores-Ramos, Adrian Cortés-Valencia, Dalia Stern, Marion Brochier, Hugo Rodrigo Sánchez-Blas, Aura Erazo-Valle-Solís, Mildred Yazmin Chávez-Cárdenas, Pabel Miranda, Martín Lajous

**Affiliations:** ^1^ CONACYT/Center for Population Health Research, National Institute of Public Health, Mexico City, Mexico; ^2^ Center for Population Health Research, National Institute of Public Health, Cuernavaca, Mexico; ^3^ National Institute of Public Health, Mexico City, Mexico; ^4^ Centro Médico Nacional 20 de Noviembre, Instituto de Seguridad y Servicios Sociales de los Trabajadores del Estado, Mexico City, Mexico; ^5^ Instituto de Seguridad y Servicios Sociales de los Trabajadores del Estado, Mexico City, Mexico; ^6^ Center for Population Health Research, National Institute of Public Health; Harvard T.H. Chan School of Public Health, Boston, MA

## Abstract

Hispanics and Indigenous women are underrepresented in cancer research. We aimed to estimate the incidence of breast cancer (BC) among indigenous and non-indigenous women and describe reproductive and lifestyle risk factors.

**METHODS:**

The baseline questionnaire was completed by 115,307 women (2006-2008). Indigenous ancestry was defined by self-adscription and/or speaking an indigenous language. Incident BC-cases were confirmed using self-reports, administrative and clinical databases, cancer registries, and death certificates. We calculated person-years from the baseline questionnaire to the date of diagnosis, death, or the end of follow-up (December 31, 2019). We age-standardized reproductive and lifestyle information.

**RESULTS:**

After a median follow-up of 10.8 years, we confirmed 1,212 BC-cases. The crude incidence rate per 100,000 person-years was 55 for indigenous and 95 for non-indigenous women; the mean age at diagnosis was 48.2 and 50.8, respectively. In indigenous BC-cases, early menarche (11.9 *v* 26.7%), first pregnancy > 25 years (36.8% *v* 51.7%), and nulliparity (11.7% *v* 14.1%) were less frequent compared to non-indigenous. The number of children (3.0 *v* 2.6) and breastfeeding > 12 months (71.6% *v* 45.2%) were higher among indigenous. Indigenous had earlier menopause (44.8 *v* 47.5 years) and more premenopausal-BC (27.8% *v* 25.1%). Oral contraceptives use (42.6% *v* 48.0%), hormone-replacement therapy (12.4% *v* 24.6%), family history of BC (11.9% *v* 15.1%) and benign breast-disease (17.8% *v* 23.0%) were less frequent in indigenous cases. Physical activity (> 150 min/week) was higher in indigenous women (34.3% *v* 27.8%). Smoking (6.6% *v* 11.2%) and alcohol consumption (47.9% *v* 61.3%) were lower in indigenous women; however, they had more diabetes (14.6% *v* 6.0%) and were at the highest tertile of the dietary glycemic index (40.2% *v* 35.0%).

**CONCLUSION:**

In the MTC, BC-incidence in indigenous women is lower than in non-indigenous; this might be explained by a lower prevalence of hormonal and reproductive risk factors and higher physical activity among indigenous women.

